# Interpolation and Amalgamation for Arrays with MaxDiff

**DOI:** 10.1007/978-3-030-71995-1_14

**Published:** 2021-03-23

**Authors:** Silvio Ghilardi, Alessandro Gianola, Deepak Kapur

**Affiliations:** 8 University of Oxford, Oxford, UK; 9 Sorbonne Université - LIP6, Paris, France; 10 Dipartimento di Matematica, Università degli Studi di Milano, Milano, Italy; 11 Faculty of Computer Science, Free University of Bozen-Bolzano, Bolzano, Italy; 12 Department of Computer Science, University of New Mexico, Albuquerque, USA

**Keywords:** Interpolation, Arrays, Amalgamation, SMT

## Abstract

In this paper, the theory of McCarthy’s extensional arrays enriched with a maxdiff operation (this operation returns the biggest index where two given arrays differ) is proposed. It is known from the literature that a diff operation is required for the theory of arrays in order to enjoy the Craig interpolation property at the quantifier-free level. However, the diff operation introduced in the literature is merely instrumental to this purpose and has only a purely formal meaning (it is obtained from the Skolemization of the extensionality axiom). Our maxdiff operation significantly increases the level of expressivity; however, obtaining interpolation results for the resulting theory becomes a surprisingly hard task. We obtain such results via a thorough semantic analysis of the models of the theory and of their amalgamation properties. The results are modular with respect to the index theory and it is shown how to convert them into concrete interpolation algorithms via a hierarchical approach.

## 1 Introduction

Since McMillan's seminal papers [31,32], interpolation has been successfully applied in software model checking, also in combination with orthogonal techniques like PDR [38] or $k$-induction [29]. The reason why interpolation techniques are so attractive is because they allow to discover in a completely *automatic* way new atoms (improperly often called 'predicates') that might contribute to the construction of invariants. In fact, software model-checking problems are typically infinite state, so invariant synthesis may require introducing formulae whose search is not finitely bounded. One way to discover them is to analyze spurious error traces; for instance, if the system under examination (described by a transition formula $Tr(\underline{x}, \underline{x}'))$ cannot reach in $n$-step an error configuration in $U(\underline{x})$ starting from an initial configuration in $In(\underline{x})$, this means that the formula

$$In(\underline{x}_0) \wedge Tr(\underline{x}_0, \underline{x}_1) \wedge \cdots \wedge Tr(\underline{x}_{n-1}, \underline{x}_n) \wedge U(\underline{x}_n)$$

⋆ The third author has been partially supported by the National Science Foundation CCF award 1908804.

is inconsistent (modulo a suitable theory $T$). From the inconsistency proof, by computing an interpolant, say at the $i$-th iteration, one can produce a formula $\phi(\underline{x})$ such that, modulo $T$, we have

$$In(\underline{x}_0) \wedge \bigwedge_{j=0}^{i} Tr(\underline{x}_{j-1}, \underline{x}_j) \models \phi(\underline{x}_i) \quad \text{and} \quad \phi(\underline{x}_i) \wedge \bigwedge_{j=i+1}^{n} Tr(\underline{x}_{j-1}, \underline{x}_j) \wedge U(\underline{x}_n) \models \bot. \tag{1}$$

This formula (and the atoms it contains) can contribute to the refinement of the current candidate loop invariant guaranteeing safey. This fact can be exploited in very different ways during invariant search, depending on the various techniques employed. It should be noticed however that interpolants are not unique and that different interpolation algorithms may return interpolants of different quality: all interpolants restrict search, but not all of them might be conclusive.

This new application of interpolation is different from the role of interpolants for analyzing proof theories of various logics starting with the pioneering works of [15,24,34]. It should be said however that Craig interpolation theorem in first order logic does not give by itself any information on the shape the interpolant can have when a specific theory is involved. Nevertheless, this is crucial for the applications: when we extract an interpolant from a trace like (1), we are typically handling a theory which might be undecidable, but whose quantifier-free fragment is decidable for satisfiability (usually within a somewhat 'reasonable' computational complexity). Thus, it is desirable (although not always possible) that the interpolant is quantifier-free, a fact which is not guaranteed in the general case. This is why a lot of effort has been made in analyzing *quantifier-free* interpolation, also exploiting its connection to semantic properties like amalgamation and strong amalgamation (see [9] for comprehensive results in the area).

The specific theories we want to analyze in this paper are variants of *McCarthy's theory of arrays* [30] *with extensionality* (see Section 3 below for a detailed description). The main operations considered in this theory are the *write* operation (i.e. the array update) and the *read* operation (i.e., the access to the content of an array cell). As such, this theory is suitable to formalize programs over arrays, like standard copying, comparing, searching, sorting, etc. functions; verification problems of this kind are collected in the SV-COMP benchmarks category "ReachSafety-Arrays"[4], where safety verification tasks involving arrays of *finite but unknown length* are considered.

By itself, the theory of arrays with extensionality does not have quantifier free interpolation [28][5]; however, in [8] it was shown that quantifier-free interpolation is restored if one enriches the language with a binary function skolemizing the extensionality axiom (the result was confirmed - via different interpolation algorithms - in [23,37]). Such a Skolem function, applied to two array variables

[4] https://sv-comp.sosy-lab.org/2020/benchmarks.php

[5] This is the counterexample (due to R. Jhala): the formula $x = wr(y, i, e)$ is inconsistent with the formula $rd(x, j) \neq rd(y, j) \wedge rd(x, k) \neq rd(y, k) \wedge j \neq k$, but all possible interpolants require quantifiers to be written (with diff symbols, instead, it is possible to write down an interpolant without quantifiers, as shown in [8]).

$a, b$, returns an index $\texttt{diff}(a, b)$ where $a, b$ differ (it returns an arbitrary value if $a$ is equal to $b$). This semantics for the `diff` operation is very undetermined and does not have a significant interpretation in concrete programs. That is why we propose to modify it in order to give it a defined and natural meaning: we ask for $\texttt{diff}(a, b)$ to return the *biggest* index where $a, b$ differ (in case $a = b$ we ask for $\texttt{diff}(a, b)$ to be the minimum index 0). Since it is natural to view arrays as functions defined on initial intervals of the nonnegative integers, this choice has a clear semantic motivation. The expressive power of the theory of arrays so enriched becomes bigger: for instance, if we also add to the language a constant symbol $\epsilon$ for the undefined array constantly equal to some 'undefined' value $\perp$ (where $\perp$ is meant to be different from the values $a[i]$ actually in use), then we can define $|a|$ as $\texttt{diff}(a, \epsilon)$. In this way we can model the fact that $a$ is undefined outside the interval $[\,0, |a|\,]$ - this is useful to formalize the above mentioned SV-COMP benchmarks.

The effectiveness of quantifier-free interpolation in the theory of arrays with maxdiff is exemplified in the simple example of Figure 1: the invariant certifying the assert in line 7 of the `Strcpy` algorithm can be obtained taking a suitable quantifier-free interpolant out of the spurious trace (1) already for $n = 2$. In more realistic examples, as witnessed by current research [2,3,4,5,16,22,25,13], it is quite clear that useful invariants require universal quantifiers to be expressed and if undecidable fragments are invaded, incomplete solvers must be used. However, even in such circumstances, quantifier-free interpolation does not lose its interest: for instance, the tool Booster [5][6] synthesizes universally quantified invariants out of quantifer-free interpolants (quantifier-free interpolation problems are generated by negating and skolemizing universally quantified formulae arising during invariants search, see [4] for details).

```
1 int a[N];
2 int b[N];
3 int I = 0;
4 while I < N do
5 |   b[I] = a[I];
6 |   I ++;
7 assert(a = b);
```

– $In(a, b, I) \equiv I = 0 \wedge |a| = N-1 \wedge |b| = N-1 \wedge N > 0$
– $Tr(a, b, I, a', b', I') \equiv I < N \wedge I' = I + 1 \wedge a' = a \wedge b' = wr(b, I, rd(a, I))$
– $U(a, b) \equiv a \neq b \wedge I = N$

**Fig. 1.** `Strcpy` function: code and associated transition system (with program counter missed in the latter for simplicity).
Loop invariant: $a = b \vee (N > \texttt{diff}(a, b) \wedge \texttt{diff}(a, b) \geq I)$.

Proving that the theory of arrays with the above 'maxdiff' operation enjoys quantifier-free interpolation revealed to be a surprisingly difficult task. In

[6] Booster is no longer maintained, however it is still referred to in current experimental evaluations [16,13].

the end, the interpolation algorithm we obtain resembles the interpolation algorithms generated via the hierarchic locality techniques introduced in [35,36] and employed also in [37]; however, its correctness, completeness and termination proofs require a large détour going through non-trivial model-theoretic arguments (these arguments do not substantially simplify adopting the complex framework of 'amalgamation closures' and '$W$-separability' of [37], and that is the reason why we preferred to supply direct proofs).

This paper concentrates on theoretical and methodological results, rather than on experimental aspects. It is almost completely dedicated to the correctness and completeness poof of our interpolation algorithm: in Subsection 3.1 we summarize our proof plan and supply basic intuitions. The paper is structured as follows: in Section 2 we recall some background, in Section 3 we introduce our theory of arrays with maxdiff; Sections 4 and 5 supply the semantic proof of the amalgamation theorem; Sections 6 and 7 are dedicated to the algorithmic aspects, whereas Section 8 analyzes complexity for the restricted case where indexes are constrained by the theory of total orders. In the final Section 9, we mention some still open problems. The main results in the paper are Theorems 2,4,5: for space reasons, *all proofs of these theorems will be only sketched*, full details are nevertheless supplied in the online available extended version [21]. This extended version contains additional material on complexity analysis and implementation. It contains also a proof about nonexistence of uniform interpolants (see [26,27,20,10,11,12] for the definition and more information on uniform interpolants).

## 2 Formal Preliminaries

We assume the usual syntactic (e.g., signature, variable, term, atom, literal, formula, and sentence) and semantic (e.g., structure, sub-structure, truth, satisfiability, and validity) notions of (possibly many-sorted) first-order logic. The equality symbol "=" is included in all signatures considered below. Notations like $E(\underline{x})$ mean that the expression (term, literal, formula, etc.) $E$ contains free variables only from the tuple $\underline{x}$. A 'tuple of variables' is a list of variables without repetitions and a 'tuple of terms' is a list of terms (possibly with repetitions). Finally, whenever we use a notation like $E(\underline{x}, \underline{y})$ we implicitly assume not only that both the $\underline{x}$ and the $\underline{y}$ are pairwise distinct, but also that $\underline{x}$ and $\underline{y}$ are disjoint. A *constraint* is a conjunction of literals. A formula is *universal* (*existential*) iff it is obtained from a quantifier-free formula by prefixing it with a string of universal (existential, resp.) quantifiers.

*Theories and satisfiability modulo theory.* A *theory* $T$ is a pair $(\Sigma, Ax_T)$, where $\Sigma$ is a signature and $Ax_T$ is a set of $\Sigma$-sentences, called the *axioms* of $T$ (we shall sometimes write directly $T$ for $Ax_T$). The *models* of $T$ are those $\Sigma$-structures in which all the sentences in $Ax_T$ are true. A $\Sigma$-formula $\phi$ is $T$*-satisfiable* (or $T$-consistent) if there exists a model $\mathcal{M}$ of $T$ such that $\phi$ is true in $\mathcal{M}$ under a suitable assignment $\mathtt{a}$ to the free variables of $\phi$ (in symbols, $(\mathcal{M}, \mathtt{a}) \models \phi$); it is *$T$-valid* (in symbols, $T \vdash \varphi$) if its negation is $T$-unsatisfiable or, equivalently, $\varphi$ is provable from the axioms of $T$ in a complete calculus for first-order logic. A theory $T = (\Sigma, Ax_T)$ is *universal* iff all sentences in $Ax_T$ are universal. A formula $\varphi_1$ *$T$-entails* a formula $\varphi_2$ if $\varphi_1 \to \varphi_2$ is *$T$-valid* (in symbols, $\varphi_1 \vdash_T \varphi_2$ or simply $\varphi_1 \vdash \varphi_2$ when $T$ is clear from the context). If $\Gamma$ is a set of formulæ and $\phi$ a formula, $\Gamma \vdash_T \phi$ means that there are $\gamma_1, \dots, \gamma_n \in \Gamma$ such that $\gamma_1 \wedge \cdots \wedge \gamma_n \vdash_T \phi$. The *satisfiability modulo the theory* $T$ (SMT($T$)) *problem* amounts to establishing the $T$-satisfiability of quantifier-free $\Sigma$-formulæ (equivalently, the $T$-satisfiability of $\Sigma$-constraints). A theory $T$ admits *quantifier-elimination* iff for every formula $\phi(\underline{x})$ there is a quantifier-free formula $\phi'(\underline{x})$ such that $T \vdash \phi \leftrightarrow \phi'$.

Some theories have special names, which are becoming standard in SMT-literature; for instance, $\mathcal{EUF}(\Sigma)$ is the pure equality theory in the signature $\Sigma$ (this is commonly abbreviated as $\mathcal{EUF}$ if there is no need to specify the signature $\Sigma$). More standard theory names will be recalled during the paper.

*Embeddings and sub-structures* The support of a structure $\mathcal{M}$ is denoted with $|\mathcal{M}|$. For a (sort, function, relation) symbol $\sigma$, we denote as $\sigma^{\mathcal{M}}$ the interpretation of $\sigma$ in $\mathcal{M}$. An embedding is a homomorphism that preserves and reflects relations and operations (see, e.g., [14]). Formally, a *$\Sigma$-embedding* (or, simply, an embedding) between two $\Sigma$-structures $\mathcal{M}$ and $\mathcal{N}$ is any mapping $\mu : |\mathcal{M}| \longrightarrow |\mathcal{N}|$ satisfying the following three conditions: (a) it is a (sort-preserving) injective function; (b) it is an algebraic homomorphism, that is for every $n$-ary function symbol $f$ and for every $a_1, \dots, a_n \in |\mathcal{M}|$, we have $f^{\mathcal{N}}(\mu(a_1), \dots, \mu(a_n)) = \mu(f^{\mathcal{M}}(a_1, \dots, a_n))$; (c) it preserves and reflects predicates, i.e. for every $n$-ary predicate symbol $P$, we have $(a_1, \dots, a_n) \in P^{\mathcal{M}}$ iff $(\mu(a_1), \dots, \mu(a_n)) \in P^{\mathcal{N}}$. If $|\mathcal{M}| \subseteq |\mathcal{N}|$ and the embedding $\mu : \mathcal{M} \longrightarrow \mathcal{N}$ is just the identity inclusion $|\mathcal{M}| \subseteq |\mathcal{N}|$, we say that $\mathcal{M}$ is a *substructure* of $\mathcal{N}$ or that $\mathcal{N}$ is a *superstructure* of $\mathcal{M}$. As it is known, the truth of a universal (resp. existential) sentence is preserved through substructures (resp. superstructures).

*Combinations of theories.* A theory $T$ is *stably infinite* iff every $T$-satisfiable quantifier-free formula (from the signature of $T$) is satisfiable in an infinite model of $T$. By compactness, it is possible to show that $T$ is stably infinite iff every model of $T$ embeds into an infinite one (see, e.g., [17]). A theory $T$ is *convex* iff for every conjunction of literals $\delta$, if $\delta \vdash_T \bigvee_{i=1}^{n} x_i = y_i$ then $\delta \vdash_T x_i = y_i$ holds for some $i \in \{1, ..., n\}$. Let $T_i$ be a stably-infinite theory over the signature $\Sigma_i$ such that the $SMT(T_i)$ problem is decidable for $i = 1, 2$ and such that $\Sigma_1$ and $\Sigma_2$ are disjoint (i.e. the only shared symbol is equality). Under these assumptions, the *Nelson-Oppen combination result* [33] says that the SMT problem for the combination $T_1 \cup T_2$ of the theories $T_1$ and $T_2$ is decidable.

*Interpolation properties.* Craig's interpolation theorem [14] roughly states that if a formula $\phi$ implies a formula $\psi$ then there is a third formula $\theta$, called an interpolant, such that $\phi$ implies $\theta$, $\theta$ implies $\psi$, and every non-logical symbol in $\theta$ occurs both in $\phi$ and $\psi$. Our interest is to specialize this result to the computation of quantifier-free interpolants modulo (combinations of) theories.

**Definition 1.** *[Plain quantifier-free interpolation] A theory $T$* admits (plain) quantifier-free interpolation *(or, equivalently,* has quantifier-free interpolants*) iff for every pair of quantifier-free formulae $\phi, \psi$ such that $\psi \wedge \phi$ is $T$-unsatisfiable, there exists a quantifier-free formula $\theta$, called an* interpolant*, such that: (i) $\psi$ $T$-entails $\theta$, (ii) $\theta \wedge \phi$ is $T$-unsatisfiable, and (iii) only the variables occurring in both $\psi$ and $\phi$ occur in $\theta$.*

In verification, the following extension of Definition 1 is considered more useful.

**Definition 2.** *[General quantifier-free interpolation] Let $T$ be a theory in a signature $\Sigma$; we say that $T$ has the* general quantifier-free interpolation property *iff for every signature $\Sigma'$ (disjoint from $\Sigma$) and for every pair of ground $\Sigma \cup \Sigma'$-formulæ $\phi, \psi$ such that $\phi \wedge \psi$ is $T$-unsatisfiable[7], there is a ground formula $\theta$ such that: (i) $\phi$ $T$-entails $\theta$; (ii) $\theta \wedge \psi$ is $T$-unsatisfiable; (iv) all relations, constants and function symbols from $\Sigma'$ occurring in $\theta$ also occur in $\phi$ and $\psi$.*

By replacing free variables with free constants, it should be clear that general quantifier-free interpolation (Definition 2) implies plain quantifier-free interpolation (Definition 1); however, the converse implication does not hold.

*Amalgamation and strong amalgamation.* Interpolation can be characterized semantically via amalgamation.

**Definition 3.** *A universal theory $T$ has the* amalgamation property *iff given models $\mathcal{M}_1$ and $\mathcal{M}_2$ of $T$ and a common submodel $\mathcal{A}$ of them, there exists a further model $\mathcal{M}$ of $T$ (called $T$-amalgam) endowed with embeddings $\mu_1 : \mathcal{M}_1 \longrightarrow \mathcal{M}$ and $\mu_2 : \mathcal{M}_2 \longrightarrow \mathcal{M}$ whose restrictions to $|\mathcal{A}|$ coincide.*

*A universal theory $T$ has the* strong amalgamation property *if the above embeddings $\mu_1, \mu_2$ and the above model $\mathcal{M}$ can be chosen so to satisfy the following additional condition: if, for some $m_1 \in |\mathcal{M}_1|, m_2 \in |\mathcal{M}_2|$, $\mu_1(m_1) = \mu_2(m_2)$ holds, then there exists an element $a$ in $|\mathcal{A}|$ such that $m_1 = a = m_2$.*

The first statement of the following theorem is an old result due to [6]; the second statement is proved in [9] (where it is also suitably reformulated for theories which are not universal):

**Theorem 1.** *Let $T$ be a universal theory. Then*
(i) *$T$ has the amalgamation property iff it admits quantifier-free interpolants;*
(ii) *$T$ has the strong amalgamation property iff it has the general quantifier-free interpolation property.*

We underline that, in presence of stable infiniteness, strong amalgamation is a modular property (in the sense that it transfers to signature-disjoint unions of theories), whereas amalgamation is not (see again [9] for details).

[7] By this (and similar notions) we mean that $\phi \wedge \psi$ is unsatisfiable in all $\Sigma'$-structures whose $\Sigma$-reduct is a model of $T$.

## 3 Arrays with MaxDiff

The *McCarthy theory of arrays* [30] has three sorts `ARRAY`, `ELEM`, `INDEX` (called "array", "element", and "index" sort, respectively) and two function symbols $rd$ ("read") and $wr$ ("write") of appropriate arities; its axioms are:

$$\forall y, i, e.\ \ rd(wr(y, i, e), i) = e$$
$$\forall y, i, j, e.\ \ i \neq j \to rd(wr(y, i, e), j) = rd(y, j).$$

The McCarthy theory of *arrays with extensionality* has the further axiom

$$\forall x, y. x \neq y \to (\exists i.\ rd(x, i) \neq rd(y, i)), \tag{2}$$

called the 'extensionality' axiom. The theory of arrays with extensionality is not universal and quantifier-free interpolation fails for it [28]. In [8] a variant of the McCarthy theory of arrays with extensionality, obtained by Skolemizing the axioms of extensionality, is introduced. This variant of the theory turns out to be universal and to enjoy quantifier-free interpolation. However, the Skolem function introduced in [8] is generic, here we want to make it more informative, so as to return the biggest index where two different arrays differ. To locate our contribution in the general context, we need the notion of an index theory.

**Definition 4.** *An* index theory $T_I$ *is a mono-sorted theory (let* `INDEX` *be its sort) satisfying the following conditions:*

- $T_I$ *is universal, stably infinite and has the general quantifier-free interpolation property (i.e. it is strongly amalgamable, see Theorem 1);*
- $SMT(T_I)$ *is decidable;*
- $T_I$ *extends the theory* $TO$ *of linear orderings with a distinguished element* 0.

We recall that $TO$ is the theory whose only proper symbols (beside equality) are a binary predicate $\leq$ and a constant 0 subject to the axioms saying that $\leq$ is reflexive, transitive, antisymmetric and total (the latter means that $i \leq j \vee j \leq i$ holds for all $i, j$). Thus, the signature of an index theory $T_I$ contains at least the binary relation symbol $\leq$ and the constant 0. In the paper, by a $T_I$-term, $T_I$-atom, $T_I$-formula, etc. we mean a term, atom, formula in the signature of $T_I$. Below, we use the abbreviation $i < j$ for $i \leq j \wedge i \neq j$. The constant 0 is meant to separate 'formally positive' indexes - those satisfying $0 \leq i$ - from the remaining 'formally negative' ones.

Examples of index theories are $TO$ itself, integer difference logic $\mathcal{IDL}$, integer linear arithmetic $\mathcal{LIA}$, and real linear arithmetics $\mathcal{LRA}$. In order to match the requirements of Definition 4, one must however make a careful choice of the language, see [9] for details: the most important detail is that integer (resp. real) division by all positive integers should be added to the language of $\mathcal{LIA}$ (resp. $\mathcal{LRA}$). For most applications, $\mathcal{IDL}$ (namely the theory of integer numbers with 0, ordering, successor and predecessor) [8] suffices as in this theory one can model counters for scanning arrays.

[8] The name 'integer difference logic' comes from the fact that atoms in this theory are equivalent to formulæ of the kind $S^n(i) \bowtie j$ (where $\bowtie \in \{\leq, \geq, =\}$), thus they represent difference bound constraints of the kind $j - i \bowtie n$ for $n \geq 0$.

Given an index theory $T_I$, we now introduce our *array theory with maxdiff* $\mathcal{ARD}(T_I)$ (parameterized by $T_I$) as follows. We still have three sorts $\mathtt{ARRAY}, \mathtt{ELEM}, \mathtt{INDEX}$; the language includes the symbols of $T_I$, the read and write operations $rd, wr$, a binary function $\mathtt{diff}$ of type $\mathtt{ARRAY} \times \mathtt{ARRAY} \to \mathtt{INDEX}$, as well as constants $\epsilon$ and $\bot$ of sorts $\mathtt{ARRAY}$ and $\mathtt{ELEM}$, respectively. The constant $\bot$ models an undetermined (e.g. undefined, not-in-use, not coming from appropriate initialization, etc.) value and $\varepsilon$ models the totally undefined array; the term $\mathtt{diff}(x, y)$ returns the maximum index where $x$ and $y$ differ and returns 0 if $x$ and $y$ are equal. [9] Formally, the axioms of $\mathcal{ARD}(T_I)$ include, besides the axioms of $T_I$, the following ones:

$$\forall y, i, e.\ \ i \geq 0 \to rd(wr(y, i, e), i) = e \tag{3}$$

$$\forall y, i, j, e.\ \ i \neq j \to rd(wr(y, i, e), j) = rd(y, j) \tag{4}$$

$$\forall x, y.\ \ x \neq y \to rd(x, \mathtt{diff}(x, y)) \neq rd(y, \mathtt{diff}(x, y)) \tag{5}$$

$$\forall x, y, i.\ \ i > \mathtt{diff}(x, y) \to rd(x, i) = rd(y, i) \tag{6}$$

$$\forall x.\ \ \mathtt{diff}(x, x) = 0 \tag{7}$$

$$\forall x.i\ \ i < 0 \to rd(x, i) = \bot \tag{8}$$

$$\forall i.\ \ rd(\varepsilon, i) = \bot \tag{9}$$

In the read-over-write axiom (3), we put the proviso $i \geq 0$ because we want all our arrays to be undefined on negative indexes (negative updates makes no sense and have no effect: by axiom (8), reading a negative index always produces $\bot$).

We call $\mathcal{AR}_{\mathrm{ext}}(T_I)$ (the 'theory of arrays with extensionality parameterized by $T_I$') the theory obtained from $\mathcal{ARD}(T_I)$ by removing the symbol $\mathtt{diff}$ and by replacing the axioms (5)-(7) by the extensionality axiom (2). Since the extensionality axioms follows from axiom (5), $\mathcal{ARD}(T_I)$ is an extension of $\mathcal{AR}_{\mathrm{ext}}(T_I)$.

As an effect of the above axioms, we have that an array $x$ is undefined outside the interval $[0, |x|]$, where $|x|$ is defined as $|x| := \mathtt{diff}(x, \varepsilon)$. Typically, this interval is finite and in fact our proof of Theorem 3 below shows that any satisfiable constraint is satisfiable in a model where all such intervals (relatively to the variables involved in the constraint) are finite.

The next lemma is immediate from the axiomatization of $\mathcal{ARD}(T_I)$:

**Lemma 1.** *An atom of the form $a = b$ is equivalent (modulo $\mathcal{ARD}(T_I)$) to*

$$\mathtt{diff}(a, b) = 0 \wedge rd(a, 0) = rd(b, 0)\ . \tag{10}$$

*An atom of the form $a = wr(b, i, e)$ is equivalent (modulo $\mathcal{ARD}$) to*

$$(i \geq 0 \to rd(a, i) = e)\ \wedge\ \forall h\ (h \neq i \to rd(a, h) = rd(b, h))\ . \tag{11}$$

*An atom of the form $\mathtt{diff}(a, b) = i$ is equivalent (modulo $\mathcal{ARD}(T_I)$) to*

$$i \geq 0\ \wedge\ \forall h\ (h > i \to rd(a, h) = rd(b, h))\ \wedge\ (i > 0 \to rd(a, i) \neq rd(b, i))\ . \tag{12}$$

[9] Notice that it might well be the case that $\mathtt{diff}(x, y) = 0$ for different $x, y$, but in that case 0 is the only index where $x, y$ differ.

For our interpolation algorithm in Section 7, we need to introduce iterated $\mathtt{diff}$ operations, similarly to [37]. As we know $\mathtt{diff}(a, b)$ returns the biggest index where $a$ and $b$ differ (it returns 0 if $a = b$). Now we want an operator that returns the last-but-one index where $a, b$ differ (0 if $a, b$ differ in at most one index), an operator that returns the last-but-two index where $a, b$ differ (0 is they differ in at most two indexes), etc. Our language is already enough expressive for that, so we can introduce such operators explicitly as follows. Given array variables $a, b$, we define by mutual recursion the sequence of array terms $b_1, b_2, \dots$ and of index terms $\mathtt{diff}_1(a, b), \mathtt{diff}_2(a, b), \dots$:

$$b_1 := b; \qquad \mathtt{diff}_1(a, b) := \mathtt{diff}(a, b_1);$$
$$b_{k+1} := wr(b_k, \mathtt{diff}_k(a, b), rd(a, \mathtt{diff}_k(a, b))); \quad \mathtt{diff}_{k+1}(a, b) := \mathtt{diff}(a, b_{k+1})$$

Intuitively, $b_{k+1}$ is the same as $b$ except for all $k$-last indexes on which $a$ and $b$ differ, in correspondence of which $b_{k+1}$ has the same value as $a$. A useful fact is that conjunctions of formulae of the kind $\bigwedge_{j<l} \mathtt{diff}_j(a, b) = k_j$ can be eliminated in favor of universal clauses in a language whose only symbol for array variables is $rd$. In detail:

**Lemma 2.** *A formula like*

$$\mathtt{diff}_1(a, b) = k_1 \wedge \cdots\cdots \wedge \mathtt{diff}_l(a, b) = k_l \tag{13}$$

*is equivalent modulo $\mathcal{ARD}$ to the conjunction of the following five formulae:*

$$k_1 \geq k_2 \wedge \cdots \wedge k_{l-1} \geq k_l \wedge k_l \geq 0 \tag{14}$$
$$\bigwedge_{j<l}(k_j > k_{j+1} \to rd(a, k_j) \neq rd(b, k_j)) \tag{15}$$
$$\bigwedge_{j<l}(k_j = k_{j+1} \to k_j = 0) \tag{16}$$
$$\bigwedge_{j\leq l}(rd(a, k_j) = rd(b, k_j) \to k_j = 0) \tag{17}$$
$$\forall h\ (h > k_l \to rd(a, h) = rd(b, h) \vee h = k_1 \vee \cdots \vee h = k_{l-1}) \tag{18}$$

### 3.1 Our roadmap

The main result of the paper is that, for every index theory $T_I$, the array theory with maxdiff $\mathcal{ARD}(T_I)$ indexed by $T_I$ *enjoys quantifier-free interpolation* and that *interpolants can be computed hierarchically* by relying on a black-box quantifier-free interpolation algorithm for the weaker theory $T_I \cup \mathcal{EUF}$ (the latter theory has quantifier free interpolation because $T_I$ is strongly amalgamable and because of Theorem 1). In this subsection, we supply intuitions and we give a qualitative high-level view to our proofs: more technical details and full proofs can be found in [21].

**The algorithm.**

By general easy transformations (recalled in Section 7 below), it is sufficient to be able to extract a quantifier-free interpolant out of a pair of quantifier-free formulae $A, B$ such that (i) $A \wedge B$ is $\mathcal{ARD}(T_I)$-inconsistent; (ii) both $A$ and $B$ are conjunctions of flat literals, i.e. of literals which are equalities between variables, disequalities between variables or literals of the form $R(\underline{x}), \neg R(\underline{x}), f(\underline{x}) = y$ (where $\underline{x}, y$ are variables, $R$ is a predicate symbol and $f$ a function symbol).

Let us call *common* the variables occurring in both $A$ and $B$. The fact that a quantifier-free interpolant exists intuitively means that there are two reasoners (an $A$-reasoner operating on formulae involving only the variables occurring in $A$ and a $B$-reasoner operating on formulae involving only the variables occurring in $B$) that are able to discover the inconsistency of $A \wedge B$ by exchanging information on the common language, i.e. by communicating each other only the entailed quantifier-free formulae involving the common variables.

A problem that can be addressed when designing an interpolation algorithm, is that there are infinitely many common terms that can be built up out of finitely many common variables and it may happen that some uncommon terms can be recognized to be equal to some common terms during the deductions performed by the $A$-reasoner and the $B$-reasoner.

As an example, suppose that $A$ contains the literals $c_1 = wr(c_2, i, e), c_1 \neq c_2, a = wr(c_3, i, e)$, where only $c_1, c_2, c_3$ are common (i.e. only these variables occur in $B$). Then using diff operations, we can deduce $i = \mathtt{diff}(c_1, c_2), e = rd(c_1, i)$ so that in the end we can conclude that $a$ is also 'common', being definable in term of common variables. Thus, the $A$-reasoner must communicate (via a defining common term or in some other indirect way) to the $B$-reasoner any fact it discovers about $a$, although $a$ was not listed among the common variables since the very beginning. In more sophisticated examples, iterated diff operations are needed to discover 'hidden' common facts.

To cope with the above problem, our algorithm *gives names* $i_k = \mathtt{diff}_k(c_1, c_2)$ to all the iterated diffs of common array variables $c_1, c_2$ (the newly introduced names $i_k$ are considered common and can be replaced back with their defining terms when the interpolants are computed at the end of the algorithm).

The second component of our algorithm is *instantiation*. Both the $A$- and the $B$-reasoner use the content of Lemmas 1 and 2 in order to handle atoms of the kind $a = b$, $a_1 = wr(a_2, i, e)$, $i = \mathtt{diff}_k(a_1, a_2)$. Whenever they come across such atoms, the equivalent formulæ supplied by these lemmas are taken into consideration; in fact, whenever the lemmas produce universally quantified clauses of the kind $\forall h\, C$, they replace in $C$ the universally quantified index variable $h$ by *all possible instantiations* with their own index terms (these are the terms built up from index variables occurring in $A$ for the $A$-reasoner and occurring in $B$ for the $B$-reasoner respectively). Such instantiations can be read as *clauses in the language of* $T_I \cup \mathcal{EUF}$ if we replace every array variable $a$ by a fresh unary function symbol $f_a$ and read terms like $rd(a, i)$ as $f_a(i)$.

Of course both the production of names for iterated diff-terms and the instantiation with owned index terms need to be repeated (possibly, infinitely many times); we prove however (this is the content of our main Theorem 4 below) that *if* $A \wedge B$ *is* $\mathcal{ARD}(T_I)$*-inconsistent, then sooner or later the union of the sets of the clauses deduced by the* $A$*-reasoner and the* $B$*-reasoner in the restricted*

*signature of* $T_I \cup \mathcal{EUF}$ *is* $T_I \cup \mathcal{EUF}$*-inconsistent*, i.e., the instantiation process terminates. This means that an interpolant can be extracted, using a black-box quantifier-free interpolation algorithm for the weaker theory $T_I \cup \mathcal{EUF}$. In the simple case where $T_I$ is just the theory $TO$ of total orders, we shall prove in Section 8 that a *quadratic* number of instantiations always suffices. In the general case, however, the situation is similar to the statement of Herbrand theorem: finitely many instantiations suffice to get an inconsistency proof in the weaker logical formalism, but a bound cannot be given.

**The proof.**

Theorem 4 is proved in a contrapositive way: we show that *if a* $T_I \cup \mathcal{EUF}$*-inconsistency never arises, then* $A \wedge B$ *is* $\mathcal{ARD}(T_I)$*-consistent*. This is proved in two steps: if $T_I \cup \mathcal{EUF}$-inconsistency does not arise, we produce two $\mathcal{ARD}(T_I)$-models $\mathcal{A}$ and $\mathcal{B}$, where $\mathcal{A}$ satisfies $A$ and $\mathcal{B}$ satisfies $B$. Moreover, $\mathcal{A}$ and $\mathcal{B}$ are built up in such a way that they share the same $\mathcal{ARD}(T_I)$-substructure. In the second step, we prove the amalgamation theorem for $\mathcal{ARD}(T_I)$, so that the amalgamated model will produce the desired model of $A \wedge B$. In fact, the two steps are inverted in our exposition: we first prove the amalgamation theorem in Section 5 (Theorem 2) and then our main theorem in Section 7 (Theorem 4).

## 4 Embeddings

We preliminarily discuss the class of models of $\mathcal{ARD}(T_I)$ and we make important clarifications about embeddings between such models. A model $\mathcal{M}$ of $\mathcal{AR}_{\text{ext}}(T_I)$ or of $\mathcal{ARD}(T_I)$ is *functional* when the following conditions are satisfied:

(i) $\texttt{ARRAY}^{\mathcal{M}}$ is a subset of the set of all positive-support functions from $\texttt{INDEX}^{\mathcal{M}}$ to $\texttt{ELEM}^{\mathcal{M}}$ (a function $a$ is *positive-support* iff $a(i) = \bot$ for every $i < 0$);

(ii) $rd$ is function application;

(iii) $wr$ is the point-wise update operation (i.e., for $i \geq 0$, the function $wr(a, i, e)$ returns the same values as the function $a$, except at the index $i$ where it returns the element $e$).

Because of the extensionality axiom, it can be shown that every model is *isomorphic to a functional one*. For an array $a \in \texttt{INDEX}^{\mathcal{M}}$ in a functional model $\mathcal{M}$ and for $i \in \texttt{INDEX}^{\mathcal{M}}$, since $a$ is a function, we interchangeably use the notations $a(i)$ and $rd(a, i)$. A functional model $\mathcal{M}$ is said to be *full* iff $\texttt{ARRAY}^{\mathcal{M}}$ consists of *all* the positive-support functions from $\texttt{INDEX}^{\mathcal{M}}$ to $\texttt{ELEM}^{\mathcal{M}}$.

Let $a, b$ be elements of $\texttt{ARRAY}^{\mathcal{M}}$ in a model $\mathcal{M}$. We say that *a and b are cardinality dependent* (in symbols, $\mathcal{M} \models \|a - b\| < \omega$) iff $\{i \in \texttt{INDEX}^{\mathcal{M}} \mid \mathcal{M} \models rd(a, i) \neq rd(b, i)\}$ is finite. Cardinality dependency in $\mathcal{M}$ is obviously an equivalence relation, that we sometimes denote as $\sim_{\mathcal{M}}$.

Passing to $\mathcal{ARD}(T_I)$, a further remark is in order: in a functional model $\mathcal{M}$ of $\mathcal{ARD}(T_I)$, the index $\texttt{diff}(a, b)$ (if it exists) is uniquely determined: it must be the maximum index where $a, b$ differ (it is 0 if $a = b$). We say that $\texttt{diff}(a, b)$ is *defined* iff there is a maximum index where $a, b$ differ (or if $a = b$).

An embedding $\mu : \mathcal{M} \longrightarrow \mathcal{N}$ between $\mathcal{AR}_{\text{ext}}(T_I)$-models is said to be $\mathtt{diff}$-faithful iff whenever $\mathtt{diff}(a, b)$ is defined so is $\mathtt{diff}(\mu(a), \mu(b))$ and it is equal to $\mu(\mathtt{diff}(a, b))$. Since there might not be a maximum index where $a, b$ differ, in principle it is not always possible to expand a functional model of $\mathcal{AR}_{\text{ext}}(T_I)$ to a functional model of $\mathcal{ARD}(T_I)$, keeping the set of indexes unchanged. Indeed, in order to do that in a $\mathtt{diff}$-faithful way, one needs to explicitly add to $\mathtt{INDEX}^{\mathcal{M}}$ new indexes including at least indexes representing the missing maximum indexes where two given array differ. This idea is used in the following lemma (proved in the online available extended version [21]):

**Lemma 3.** *For every index theory $T_I$, every model of $\mathcal{AR}_{\text{ext}}(T_I)$ has a $\mathtt{diff}$-faithful embedding into a model of $\mathcal{ARD}(T_I)$.*

## 5 Amalgamation

We now sketch the proof of the amalgamation property for $\mathcal{ARD}(T_I)$. We recall that strong amalgamation holds for models of $T_I$ (see Definition 4).

**Theorem 2.** *$\mathcal{ARD}(T_I)$ enjoys the amalgamation property.*

*Proof.* Take two embeddings $\mu_1 : \mathcal{N} \longrightarrow \mathcal{M}_1$ and $\mu_2 : \mathcal{N} \longrightarrow \mathcal{M}_2$. As we know, we can suppose—w.l.o.g.—that $\mathcal{N}, \mathcal{M}_1, \mathcal{M}_2$ are functional models; in addition, via suitable renamings, we can freely suppose that $\mu_1, \mu_2$ restricts to inclusions for the sorts INDEX and ELEM, and that $(\mathtt{ELEM}^{\mathcal{M}_1} \setminus \mathtt{ELEM}^{\mathcal{N}}) \cap (\mathtt{ELEM}^{\mathcal{M}_2} \setminus \mathtt{ELEM}^{\mathcal{N}}) = \emptyset$, $(\mathtt{INDEX}^{\mathcal{M}_1} \setminus \mathtt{INDEX}^{\mathcal{N}}) \cap (\mathtt{INDEX}^{\mathcal{M}_2} \setminus \mathtt{INDEX}^{\mathcal{N}}) = \emptyset$. To build the amalgamated model of $\mathcal{ARD}(T_I)$, we first build a full model $\mathcal{M}$ of $\mathcal{AR}_{\text{ext}}(T_I)$ with $\mathtt{diff}$-faithful embeddings $\nu_1 : \mathcal{M}_1 \longrightarrow \mathcal{M}$ and $\nu_2 : \mathcal{M}_2 \longrightarrow \mathcal{M}$ such that $\nu_1 \circ \mu_1 = \nu_2 \circ \mu_2$. If we succeed, the claim follows by Lemma 3: indeed, thanks to that lemma, we can embed in a $\mathtt{diff}$-faithful way $\mathcal{M}$ (which is a model of $\mathcal{AR}_{\text{ext}}(T_I)$) to a model $\mathcal{M}'$ of $\mathcal{ARD}(T_I)$, which is the required $\mathcal{ARD}(T_I)$-amalgam.

We take the $T_I$-reduct of $\mathcal{M}$ to be a model supplied by the strong amalgamation property of $T_I$ (again, we can freely assume that the $T_I$-reducts of $\mathcal{M}_1, \mathcal{M}_2$ identically include in it); we let $\mathtt{ELEM}^{\mathcal{M}}$ to be $\mathtt{ELEM}^{\mathcal{M}_1} \cup \mathtt{ELEM}^{\mathcal{M}_2}$. We need to define $\nu_i : \mathcal{M}_i \longrightarrow \mathcal{M}$ $(i = 1, 2)$ in such a way that $\nu_i$ is $\mathtt{diff}$-faithful and $\nu_1 \circ \mu_1 = \nu_2 \circ \mu_2$. We take the INDEX and the ELEM-components of $\nu_1, \nu_2$ to be just identical inclusions. The only relevant point is the action of $\nu_i$ on $\mathtt{ARRAY}^{\mathcal{M}_i}$: since we have strong amalgamation for indexes, in order to define it, it is sufficient to extend any $a \in \mathtt{ARRAY}^{\mathcal{M}_i}$ to all the indexes $k \in (\mathtt{INDEX}^{\mathcal{M}} \setminus \mathtt{INDEX}^{\mathcal{M}_i})$. For indexes $k \in (\mathtt{INDEX}^{\mathcal{M}} \setminus (\mathtt{INDEX}^{\mathcal{M}_1} \cup \mathtt{INDEX}^{\mathcal{M}_2}))$ we can just put $\nu_i(a)(k) = \bot$. If $k \in (\mathtt{INDEX}^{\mathcal{M}} \setminus \mathtt{INDEX}^{\mathcal{M}_i})$ and $k \in (\mathtt{INDEX}^{\mathcal{M}_1} \cup \mathtt{INDEX}^{\mathcal{M}_2})$, then $k \in (\mathtt{INDEX}^{\mathcal{M}_{3-i}} \setminus \mathtt{INDEX}^{\mathcal{N}})$; the definition for such $k$ is as follows:

(*) we let $\nu_i(a)(k)$ be equal to $\mu_{3-i}(c)(k)$, where $c$ is any array $c \in \mathtt{ARRAY}^{\mathcal{N}}$ for which there is $a' \in \mathtt{ARRAY}^{\mathcal{M}_i}$ such that $a \sim_{\mathcal{M}_i} a'$ and such that the relation $k > \mathtt{diff}^{\mathcal{M}_i}(a', \mu_i(c))$ holds in $\mathtt{INDEX}^{\mathcal{M}}$;[10] if such $c$ does not exist, then we put $\nu_i(a)(k) = \bot$.

[10] This should be properly written as $k > \nu_i(\mathtt{diff}^{\mathcal{M}_i}(a', \mu_i(c)))$, however recall that the INDEX-component of $\nu_i$ is identity, so the simplified notation is nevertheless correct.

Definition (*) is forced by some constraints that $\nu_i(a)(k)$ must satisfy. Of course, definition (*) itself needs to be justified: besides showing that it enjoys the required properties, we must also prove that it is well-given (i.e. that it does not depend on the selected $c$ and $a'$). It is easy to see that, if the definition is correct, then we have $\nu_1 \circ \mu_1 = \nu_2 \circ \mu_2$; also, it is clear that $\nu_i$ preserves read and write operations (hence, it is a homomorphism) and is injective. For (i) justifying the definition of $\nu_i$ and (ii) showing that it is also $\mathtt{diff}$-faithful, we need to show the following two claims (the proof is not easy, see the extended version [21] for details) for arrays $a_1, a_2 \in \mathtt{ARRAY}_1^{\mathcal{M}}$, for an index $k \in (\mathtt{INDEX}^{\mathcal{M}_2} \setminus \mathtt{INDEX}^{\mathcal{N}})$ and for arrays $c_1, c_2 \in \mathtt{ARRAY}^{\mathcal{N}}$ (checking the same facts in $\mathcal{M}_2$ is symmetrical):

(i) if $a_1 \sim_{\mathcal{M}_1} a_2$ and $k > \mathtt{diff}^{\mathcal{M}_1}(a_1, \mu_1(c_1))$, $k > \mathtt{diff}^{\mathcal{M}_1}(a_2, \mu_1(c_2))$, then $\mu_2(c_1)(k) = \mu_2(c_2)(k)$.

(ii) if $k > \mathtt{diff}^{\mathcal{M}_1}(a_1, a_2)$, then $\nu_1(a_1)(k) = \nu_1(a_2)(k)$. $\dashv$

## 6 Satisfiability

The key step of the interpolation algorithm that will be proposed in Section 7 depends upon the problem of checking satisfiability (modulo $\mathcal{ARD}(T_I)$) of quantifier-free formulæ; this will be solved in the present section by adapting instantiation techniques, like those from [7].

We define the *complexity* $c(t)$ of a term $t$ as the number of function symbols occurring in $t$ (thus variables and constants have complexity 0). A *flat* literal $L$ is a formula of the kind $x_1 = t$ or $x_1 \neq x_2$ or $R(x_1, \ldots, x_n)$ or $\neg R(x_1, \ldots, x_n)$, where the $x_i$ are variables, $R$ is a relation symbol, and $t$ is a term of complexity less or equal to 1. If $\mathcal{I}$ is a set of $T_I$-terms, an *$\mathcal{I}$-instance* of a universal formula of the kind $\forall i\, \phi$ is a formula of the kind $\phi(t/i)$ for some $t \in \mathcal{I}$.

A pair of sets of quantifier-free formulae $\Phi = (\Phi_1, \Phi_2)$ is a *separated pair* iff

(1) $\Phi_1$ contains equalities of the form $\mathtt{diff}_k(a, b) = i$ and $a = wr(b, i, e)$; moreover if it contains the equality $\mathtt{diff}_k(a, b) = i$, it must also contain an equality of the form $\mathtt{diff}_l(a, b) = j$ for every $l < k$;

(2) $\Phi_2$ contains Boolean combinations of $T_I$-atoms and of atoms of the forms:

$$rd(a, i) = rd(b, j), \quad rd(a, i) = e, \quad e_1 = e_2, \tag{19}$$

where $a, b, i, j, e, e_1, e_2$ are variables or constants of the appropriate sorts.

The separated pair is said to be finite iff $\Phi_1$ and $\Phi_2$ are both finite.

In practice, in a separated pair $\Phi = (\Phi_1, \Phi_2)$, reading $rd(a, i)$ as a functional application, it turns out that *the formulæ from $\Phi_2$ can be translated into quantifier-free formulæ of the combined theory $T_I \cup \mathcal{EUF}$* (the array variables occurring in $\Phi_2$ are converted into free unary function symbols). $T_I \cup \mathcal{EUF}$ enjoys the decidability of the quantifier-free fragment and has quantifier-free interpolation because $T_I$ is an index theory (see Nelson-Oppen results [33] and Theorem 1): we adopt a hierarchical approach (similar to [35,36]) and *we rely on satisfiability and interpolation algorithms for such a theory as black boxes.*

Let $\mathcal{I}$ be a set of $T_I$-terms and let $\Phi = (\Phi_1, \Phi_2)$ be a separated pair; we let $\Phi(\mathcal{I}) = (\Phi_1(\mathcal{I}), \Phi_2(\mathcal{I}))$ be the smallest separated pair satisfying the following conditions:

- $\Phi_1(\mathcal{I})$ is equal to $\Phi_1$ and $\Phi_2(\mathcal{I})$ contains $\Phi_2$;
- $\Phi_2(\mathcal{I})$ contains all $\mathcal{I}$-instances of the two formulæ

$$\forall i\, rd(\varepsilon, i) = \bot,\ \forall i\, (i < 0 \to rd(a, i) = \bot),$$

where $a$ is any array variable occurring in $\Phi_1$ or $\Phi_2$;
- if $\Phi_1$ contains the atom $a = wr(b, i, e)$ then $\Phi_2(\mathcal{I})$ contains *all the $\mathcal{I}$-instances of the formulae* (11);
- if $\Phi_1$ contains the conjunction $\bigwedge_{i=1}^{l} \mathtt{diff}_i(a, b) = k_i$, then $\Phi_2(\mathcal{I})$ contains the formulae (14), (15), (16), (17) as well as *all $\mathcal{I}$-instances of the formula* (18).

For $M \in \mathbb{N} \cup \{\infty\}$, the *$M$-instantiation* of $\Phi = (\Phi_1, \Phi_2)$ is the separated pair $\Phi(\mathcal{I}_\Phi^M) = (\Phi_1(\mathcal{I}_\Phi^M), \Phi_2(\mathcal{I}_\Phi^M))$, where $\mathcal{I}_\Phi^M$ is the set of $T_I$-terms of complexity at most $M$ built up from the index variables occurring in $\Phi_1, \Phi_2$. The *full instantiation* of $\Phi = (\Phi_1, \Phi_2)$ is the separated pair $\Phi(\mathcal{I}_\Phi^\infty) = (\Phi_1(\mathcal{I}_\Phi^\infty), \Phi_2(\mathcal{I}_\Phi^\infty))$ (which is usually not finite). A separated pair $\Phi = (\Phi_1, \Phi_2)$ is *$M$-instantiated* iff $\Phi = \Phi(\mathcal{I}_\Phi^M)$; it is $\mathcal{ARD}(T_I)$-satisfiable iff so it is the formula $\bigwedge \Phi_1 \wedge \bigwedge \Phi_2$[11]

*Example 1. Let $\Phi_1$ contain the four atoms*

$$\{\ \mathtt{diff}(a, c_1) = i_1,\ \mathtt{diff}(b, c_2) = i_1,\ a = wr(a_1, i_3, e_3),\ a_1 = wr(b, i_1, e_1)\ \}$$

*and let $\Phi_2$ be empty. Then $(\Phi_1, \Phi_2)$ is a separated pair; 0-instantiating it adds to $\Phi_2$ the following formulae (we delete those which are redundant)*

$$\begin{array}{ll}
i_1 \geq 0 & \\
rd(a, i_1) = rd(c_1, i_1) \to i_1 = 0 & rd(b, i_1) = rd(c_2, i_1) \to i_1 = 0 \\
i_3 > i_1 \to rd(a, i_3) = rd(c_1, i_3) & i_3 > i_1 \to rd(b, i_3) = rd(c_2, i_3) \\
i_3 \geq 0 \to rd(a, i_3) = e_3 & i_1 \geq 0 \to rd(a_1, i_1) = e_1 \\
i_1 \neq i_3 \to rd(a, i_1) = rd(a_1, i_1) & i_1 \neq i_3 \to rd(a_1, i_3) = rd(b, i_3)
\end{array}$$

The following results are proved in the extended version [21]:

**Lemma 4.** *Let $\phi$ be a quantifier-free formula; then it is possible to compute finitely many finite separation pairs $\Phi^1 = (\Phi_1^1, \Phi_2^1), \ldots, \Phi^n = (\Phi_1^n, \Phi_2^n)$ such that $\phi$ is $\mathcal{ARD}(T_I)$-satisfiable iff so is one of the $\Phi^i$.*

**Lemma 5.** *The following conditions are equivalent for a finite separation pair $\Phi = (\Phi_1, \Phi_2)$:*
(i) *$\Phi$ is $\mathcal{ARD}(T_I)$-satisfiable;*
(ii) *$\bigwedge \Phi_2(\mathcal{I}_\Phi^0)$ is $T_I \cup \mathcal{EUF}$-satisfiable.*

**Theorem 3.** *The $SMT(\mathcal{ARD}(T_I))$ problem is decidable for every index theory $T_I$ (i.e. for every theory satisfying Definition 4).*

[11] This might be an infinitary formula if $\Phi$ is not finite. In such a case, satisfiability obviously means that there is a model $\mathcal{M}$ where we can assign values to all variables occurring in the formulæ from $\Phi_1 \cup \Phi_2$ in such a way that such formulæ become simultaneously true.

Concerning the complexity of the above procedure, notice that the satisfiability of the quantifier-free fragment of common index theories (like $\mathcal{IDL}$, $\mathcal{LIA}$, $\mathcal{LRA}$) is decidable in NP; as a consequence, from the above proof we get (for such index theories) also an NP bound for our $SMT(\mathcal{ARD}(T_I))$)-problems because 0-instantiation is clearly finite and polynomial. The fact that 0-instantiation suffices is a common feature of the above satisfiability procedure and of the satisfiability procedures from [7]. Unfortunately, when coming to interpolation algorithms in the next section, there is no evidence that 0-instantiation suffices.

## 7 An interpolation algorithm

Since amalgamation is equivalent to quantifier-free interpolation for universal theories like $\mathcal{ARD}(T_I)$ (see Theorem 1), Theorem 2 ensures that $\mathcal{ARD}(T_I)$ has the quantifier-free interpolation property. However, the proof of Theorem 2 is not constructive, so in order to compute an interpolant for an $\mathcal{ARD}(T_I)$-unsatisfiable conjunction like $\psi(\underline{x}, \underline{y}) \wedge \phi(\underline{y}, \underline{z})$, one should enumerate all quantifier-free formulæ $\theta(\underline{y})$ which are logical consequences of $\phi$ and are inconsistent with $\psi$ (modulo $\mathcal{ARD}(T_I)$). Since the quantifier-free fragment of $\mathcal{ARD}(T_I)$ is decidable by Theorem 3, this is an effective procedure and, since interpolants of jointly unsatisfiable pairs of formulæ exist, it also terminates. However, such kind of an algorithm is not practical.

In this section, we improve the situation by supplying a better algorithm based on instantiation (à-la-Herbrand). In the next section, using the results of the present section, for the special case where $T_I$ is just the theory of linear orders, we identify a complexity bound for this algorithm.

Our problem is the following: given two quantifier-free formulae $A$ and $B$ such that $A \wedge B$ is not satisfiable (modulo $\mathcal{ARD}(T_I)$), to compute a quantifier-free formula $C$ such that $\mathcal{ARD}(T_I) \models A \to C$, $\mathcal{ARD}(T_I) \models C \wedge B \to \bot$ and such that $C$ contains only the variables (of sort `INDEX`, `ARRAY`, `ELEM`) which occur both in $A$ and in $B$.

We call the variables occurring in both $A$ and $B$ *common variables*, whereas the variables occurring in $A$ (resp. in $B$) are called *$A$-variables* (resp. *$B$-variables*). The same terminology applies to terms, atoms and formulae: e.g., a term $t$ is an $A$-term ($B$-term, common term) iff it is built up from $A$-variables ($B$-variables, common variables, resp.).

The following operations can be freely performed (see [9] or [8] for details):

(i) pick an $A$-term $t$ and a fresh variable $a$ (of appropriate sort) and conjoin $A$ to $a = t$ ($a$ will be considered an $A$-variable from now on);

(ii) pick a $B$-term $t$ and a fresh variable $b$ (of appropriate sort) and conjoin $B$ to $b = t$ ($b$ will be considered a $B$-variable from now on);

(iii) pick a common term $t$ and a fresh variable $c$ (of appropriate sort) and conjoin both $A$ and $B$ to $c = t$ ($c$ will be considered a common variable from now on);

(iv) conjoin $A$ with some quantifier-free $A$-formula which is implied (modulo $\mathcal{ARD}(T_I)$) by $A$;

(v) conjoin $B$ with some quantifier-free $B$-formula which is implied (modulo $\mathcal{ARD}(T_I)$) by $B$.

Operations (i)-(v) either add logical consequences or explicit definitions that can be eliminated (if desired) after the final computation of the interpolant. In addition, notice that if $A$ is the form $A' \vee A''$ (resp. $B$ is of the form $B' \vee B''$) then from interpolants of $A' \wedge B$ and $A'' \wedge B$ (resp. of $A \wedge B'$ and $A \wedge B''$), we can recover an interpolant of $A \wedge B$ by taking disjunction (resp. conjunction).

Because of the above remarks, using the procedure in the proof of Lemma 4, both $A$ and $B$ are assumed to be given in the form of finite separated pairs. Thus $A$ is of the form $\bigwedge A_1 \wedge \bigwedge A_2$, $B$ is of the form $\bigwedge B_1 \wedge \bigwedge B_2$, for separated pairs $(A_1, A_2)$ and $(B_1, B_2)$. Also, by (iv)-(v) above, $A$ and $B$ are assumed to be both 0-instantiated. We call $A$ (resp. $B$) the separated pair $(A_1, A_2)$ (resp. $(B_1, B_2)$). We also use the letters $A_1, A_2, B_1, B_2$ both for sets of formulae and for the corresponding conjunctions; similarly, $A$ represent both the pair $(A_1, A_2)$ and the conjunction $\bigwedge A_1 \wedge \bigwedge A_2$ (and similarly for $B$).

The formulæ from $A_2$ and $B_2$ are formulæ from the signature of $T_I \cup \mathcal{EUF}$ (after rewriting terms of the kind $rd(a, i)$ to $f_a(i)$, where the $f_a$ are free function symbols). Of course, if $A_2 \wedge B_2$ is $T_I \cup \mathcal{EUF}$-inconsistent, *we can get our quantifier-free interpolant by using our black box algorithm for interpolation in the weaker theory* $T_I \cup \mathcal{EUF}$: recall that $T_I \cup \mathcal{EUF}$ has quantifier-free interpolation because $T_I$ is an index theory and for Theorem 1. The remarkable fact is that $A_2 \wedge B_2$ always becomes $T_I \cup \mathcal{EUF}$-inconsistent if *sufficiently many* `diffs` *among common array variables are introduced* and *sufficiently many instantiations are performed.*

Formally, we shall *apply the loop below until $A_2 \wedge B_2$ becomes inconsistent*: the loop is justified by (i)-(v) above and Theorem 4 guarantees that $A_2 \wedge B_2$ eventually becomes inconsistent modulo $T_I \cup \mathcal{EUF}$, if $A \wedge B$ was originally inconsistent modulo $\mathcal{ARD}(T_I)$. When $A_2 \wedge B_2$ becomes inconsistent modulo $T_I \cup \mathcal{EUF}$, we can get our interpolant using the interpolation algorithm for $T_I \cup \mathcal{EUF}$. [Of course, in the interpolant returned by $T_I \cup \mathcal{EUF}$, the extra variables introduced by the explicit definitions from (iii) above need to be eliminated.] We need a counter $M$ recording how many times the Loop below has been executed (initially $M = 0$).

**Loop** *(to be repeated until $A_2 \wedge B_2$ becomes inconsistent modulo $T_I \cup \mathcal{EUF}$). Pick two distinct common* `ARRAY`*-variables $c_1, c_2$ and $n \geq 1$ and s.t. no conjunct of the kind $\mathtt{diff}_n(c_1, c_2) = k$ occurs in both $A_1$ and $B_1$ for some $n \geq 1$ (but s.t. for every $l < n$ there is a conjunct of the form $\mathtt{diff}_l(a, b) = k$ occurring in both $A_1$ and $B_1$). Pick also a fresh* `INDEX` *constant $k_n$; conjoin $\mathtt{diff}_n(c_1, c_2) = k_n$ to both $A_1$ and $B_1$; then $M$-instantiate both $A$ and $B$. Increase $M$ to $M + 1$.*

Notice that the fresh index constants $k_n$ introduced during the loop are considered common constants (they come from explicit definitions like (iii) above) and so they are considered in the $M$-instantiation of both $A$ and $B$.

*Example 2. Let $A$ be the formula $\bigwedge \Phi_1$ from Example 1 and let $B$ be*

$$i_1 < i_2 \;\wedge\; i_2 < i_3 \;\wedge\; rd(c_1, i_2) \neq rd(c_2, i_2)$$

*$B$ is 0-instantiated; 0-instantiating $A$ produces the formulæ shown in Example 1. The loop needs to be executed twice; it adds the literals $\mathtt{diff}_0(c_1, c_2) =$*

*$k_0, \mathtt{diff}_1(c_1, c_2) = k_1$; 0-instantiation produces formulae $A_2$, $B_2$ whose conjunction is $T_I \cup \mathcal{EUF}$-inconsistent (inconsistency can be tested via an SMT-solver like* z3 *or* MathSat*, see the ongoing implementation [1]). The related $T_I \cup \mathcal{EUF}$-interpolant (once $k_0$ and $k_1$ are replaced by $\mathtt{diff}_0(c_1, c_2)$ and $\mathtt{diff}_1(c_1, c_2)$, respectively) gives our $\mathcal{ARD}(T_I)$-interpolant.* ⊣

**Theorem 4.** *If $A \wedge B$ is $\mathcal{ARD}(T_I)$-inconsistent, then the above loop terminates.*

*Proof.* Suppose that the loop does not terminate and let $A' = (A'_1, A'_2)$ and $B' = (B'_1, B'_2)$ be the separated pairs obtained after infinitely many executions of the loop (they are the union of the pairs obtained in each step). Notice that both $A'$ and $B'$ are fully instantiated.[12] We claim that $(A', B')$ is $\mathcal{ARD}(T_I)$-consistent (contradicting the assumption that $(A, B)$ was already $\mathcal{ARD}(T_I)$-inconsistent).

Since no contradiction was found, by compactness of first-order logic, $A'_2 \cup B'_2$ has a $T_I \cup \mathcal{EUF}$-model $\mathcal{M}$ (below we treat index and element variables occurring in $A, B$ as free constants and the array variables occurring in $A, B$ as free unary function symbols). $\mathcal{M}$ is a two-sorted structure (the sorts are INDEX and ELEM) endowed for every array variable $a$ occurring in $A, B$ of a function $a^{\mathcal{M}} : \mathtt{INDEX}^{\mathcal{M}} \longrightarrow \mathtt{ELEM}^{\mathcal{M}}$. In addition, $\mathtt{INDEX}^{\mathcal{M}}$ is a model of $T_I$. We build three $\mathcal{ARD}(T_I)$-structures $\mathcal{A}, \mathcal{B}, \mathcal{C}$ and two embeddings $\mu_1 : \mathcal{C} \longrightarrow \mathcal{A}$, $\mu_2 : \mathcal{C} \longrightarrow \mathcal{B}$ such that $\mathcal{A} \models A'$, $\mathcal{B} \models B'$ and such that for every common variable $x$ we have $\mu_1(x^{\mathcal{C}}) = x^{\mathcal{A}}$ and $\mu_2(x^{\mathcal{C}}) = x^{\mathcal{B}}$. The consistency of $A' \cup B'$ then follows from the amalgamation Theorem 2. The two structures $\mathcal{A}, \mathcal{B}$ are obtained by taking the full functional model induced by the restriction of $\mathcal{M}$ to the interpretation of $A$-terms and $B$-terms (respectively) of sort INDEX, ELEM and then by applying Lemma 3; the construction of $\mathcal{C}$ requires some subtleties, to be detailed in the extended version [21], where the full proof of the theorem is provided. ⊣

## 8 When indexes are just a total order

Comparing the results from Sections 7 and 6, a striking difference emerges: whereas variable and constant instantiations are sufficient for satisfiability checking, our interpolation algorithm requires full instantiation over all common terms. Such a full instantiation might be quite impractical, especially in index theories like $\mathcal{LIA}$ and $\mathcal{LRA}$ (it is less annoying in theories like $\mathcal{IDL}$: here all terms are of the kind $S^n(x)$ or $P^n(x)$, where $x$ is a variable or 0 and $S, P$ are the successor and the predecessor functions). The problem disappears in simpler theories like the theory of linear orders $TO$, where all terms are variables (or the constant 0). Still, even in the case of $TO$, the proof of Theorem 4 does not give a bound for termination of the interpolation algorithm: we know that sooner or later an inconsistency will occur, but we do not know how many times we need to execute the main loop. We now improve the proof of Theorem 4 by supplying the missing bound. In this section, the index theory is fixed to be $TO$ and we abbreviate $\mathcal{ARD}(TO)$ as $\mathcal{ARD}$. The full proof of the theorem below is in [21].

[12] On the other hand, the joined pair $(A'_1 \cup B'_1, A'_2 \cup B'_2)$ is not even 0-instantiated.

**Theorem 5.** *If $A \wedge B$ is inconsistent modulo $\mathcal{ARD}$, then the above loop terminates in at most $(\frac{m^2-m}{2}) \cdot (n+1)$ steps, where $n$ is the number of the index variables occurring in $A, B$ and $m$ is the number of the common array variables.*

*Proof.* We sketch a proof of the theorem: the idea is that if after $N := (\frac{m^2-m}{2}) \cdot (n+1)$ steps no inconsistency occurs, then we can run the algorithm for infinitely many further steps without finding an inconsistency either. Let $A^N = (A_1^N, A_2^N)$ and $B^N = (B_1^N, B_2^N)$ be obtained after $N$-executions of the loop and let $\mathcal{M}$ be a $\mathcal{TO} \cup \mathcal{EUF}$-model of $A_2^N \wedge B_2^N$. Fix a pair of distinct common array variables $c_1, c_2$ to be handled in Step $N+1$; since all pairs of common array variables have been examined in a fair way, $A_1^N$ and $B_1^N$ contain the atom $\mathtt{diff}_{n+1}(c_1, c_2) = k_{n+1}$ (in fact $N := (\frac{m^2-m}{2}) \cdot (n+1)$ and $(\frac{m^2-m}{2})$ is the number of distinct unordered pairs of common array variables, so the pair $(c_1, c_2)$ has been examined more than $n$ times). In $\mathcal{M}$, some index variable $k_l$ for $l \leq k_{n+1}$, if not assigned to 0, is assigned to an element $x$ which is different from the elements assigned to the $n$ variables occurring in $A, B$. This allows us to enlarge $\mathcal{M}$ to a superstructure which is a model of $A_2^{N+1} \wedge B_2^{N+1}$ by 'duplicating' $x$. Continuing in this way, we produce a chain of $\mathcal{TO} \cup \mathcal{EUF}$-models witnessing that we can run infinitely many steps of the algorithm without finding an inconsistency. $\dashv$

## 9 Conclusions and further work

We studied an extension of McCarthy theory of arrays with a maxdiff symbol. This symbol produces a much more expressive theory than the theory of plain diff symbol already considered in the literature [8,37].

We have also considered another strong enrichment, namely the combination with arithmetic theories like $\mathcal{IDL}, \mathcal{LIA}, \mathcal{LRA}, \ldots$ (all such theories are encompassed by the general notion of an 'index theory'). Such a combination is non trivial because it is a non disjoint combination (the ordering relation is in the shared signature) and does not fulfill the $T_0$-compatibility requirements of [17,19,18] needed in order to modularly import satisfiability and interpolation algorithms from the component theories.

The above enrichments come with a substantial cost: although decidability of satisfiability of quantifier-free formulae is not difficult to obtain, quantifier-free interpolation becomes challenging. In this paper, we proved that quantifier-free interpolants indeed do exist: the interpolation algorithm is indeed rather simple, but its justification comes via a complicated détour involving semantic investigations on amalgamation properties.

The interpolation algorithm is based on hierarchic reduction to general quantifier-free interpolation in the index theory. The reduction requires the introduction of iterated diff terms and a finite number of instantiations of the universal clauses associated to write and diff-atoms. For the simple case where the index theory is just the theory of total orders, we were able to polynomially bound the depth of the iterated diff terms to be introduced as well as the number of instantiations needed. The main open problem we leave for future is the determination of analogous bounds for richer index theories.
